# Mesenchymal Stromal Cells in Ischemic Brain Injury

**DOI:** 10.3390/cells11061013

**Published:** 2022-03-17

**Authors:** Beverly Brooks, Dominique Ebedes, Ahsan Usmani, Joaquin Vega Gonzales-Portillo, Daniel Gonzales-Portillo, Cesario V. Borlongan

**Affiliations:** 1 Center of Excellence for Aging and Brain Repair, University of South Florida Morsani College of Medicine, 12901 Bruce B Downs Blvd, Tampa, FL 33612, USA; beverly14@usf.edu (B.B.); debedes@usf.edu (D.E.); ausmani@usf.edu (A.U.); 2 Universidad Peruana de Ciencias Aplicadas, Prolongación Primavera 2390, Lima 15023, Peru; jovgagp95@gmail.com; 3 Department of Psychology, University of Florida, 945 Center Dr, Gainesville, FL 32611, USA; danielgp214@outlook.com

**Keywords:** mesenchymal stromal cells, mesenchymal stem cells, stroke, brain injury, stem cells

## Abstract

Ischemic brain injury represents a major cause of death worldwide with limited treatment options with a narrow therapeutic window. Accordingly, novel treatments that extend the treatment from the early neuroprotective stage to the late regenerative phase may accommodate a much larger number of stroke patients. To this end, stem cell-based regenerative therapies may address this unmet clinical need. Several stem cell therapies have been tested as potentially exhibiting the capacity to regenerate the stroke brain. Based on the long track record and safety profile of transplantable stem cells for hematologic diseases, bone marrow-derived mesenchymal stromal cells or mesenchymal stromal cells have been widely tested in stroke animal models and have reached clinical trials. However, despite the translational promise of MSCs, probing cell function remains to be fully elucidated. Recognizing the multi-pronged cell death and survival processes that accompany stroke, here we review the literature on MSC definition, characterization, and mechanism of action in an effort to gain a better understanding towards optimizing its applications and functional outcomes in stroke.

## 1. Introduction

Stroke is the leading cause of disability in the United States. Ischemic stroke has a greater prevalence, contributing to 87% of overall stroke incidents. Within hours, excitotoxicity, neuroinflammation, and cell death can contribute to damage and, conversely, recovery, in the brain [1]. Despite the growing incidence of stroke, there are limited therapeutic options available for patients [2,3]. Recombinant tissue plasminogen activator (rtPA) is the only neuroprotective agent therapy for thrombotic stroke [3]. However, this therapy has a limited clinical reach, as it has a therapeutic window of 3–4.5 h, leaving only a small percent of patients eligible for this therapy [3]. Endovascular mechanical thrombectomy can be considered an interventional treatment for patients with an acute ischemic stroke with a large vessel occlusion presenting in the anterior circulation and onset within 6 h [4]. However, despite successful intervention, disability-free survival remains low [4]. Physical therapy is an option available for stroke patients to improve mobility and motor skills which may be beneficial for patients [3]. While public stroke education has resulted in a decrease in stroke death, a significant disparity remains between patients who are able to receive stroke intervention and those who must rely on rehabilitation therapies [4]. The therapeutic focus has thus shifted to stem cell therapy as a potential bridge to address the significant incidence of disability.

A review of the definition, characterization, and mechanism of action of MSCs is crucial to gain a better understanding towards optimizing its applications and functional outcomes in stroke. An outdated definition of MSCs has led to inconsistencies in the field of research and treatment. We believe there needs to be a call for standardization of this topic moving forward. A secondary issue surrounds the diverse mechanism of action, as well as the underlying ones, of MSC. This review discusses inaccurate MSC definitions, a lack of universal standard across academia regarding MSC characterization, and the biologically complex processes completed by MSCs within applications associated with ischemic stroke outcomes. Regarding the current literature surrounding MSC application in stroke events, this review dissects errors present within said current literature that impact and circumvent further scientific growth due to the standardization and characterization issues currently present. As corresponding literature has begun to compile surrounding this topic, the novel aspect of this review pursues a greater analysis of the current literature to drive more substantial and detailed growth as the topic is pursued in more substantial quantities in the future. By providing a detailed and systemic investigation regarding stroke outcomes associated with MSC utilization, scientific and clinical communities can further combine characterizations and definitions to drive a more widely understood and discussed employment of MSC therapies.

## 2. Stem Cell-Based Regenerative Medicine

Stem cells are defined by their ability to differentiate into a variety of different cell types and continuously self-renew [5,6,7]. The application of stem cells has been of great interest in regenerative medicine regarding tissue restoration and repair. This includes their use in treatment for neurological disorders and brain injuries, such as stroke, although deciding how to transplant stem cells and what type of stem cells to use is still a significant focus of research [8]. Further classification of stem cells is primarily based on their potential for differentiation into different cell types as well as the developmental stage from which they arise [5,6,7]. Stem cells with the greatest capacity for differentiation are totipotent stem cells, so-called because of their ability to divide into all tissue types of the organism during embryological development, as well as extraembryonic tissues such as the placenta [6,7]. Pluripotent stem cells are secondary to totipotent stem cells in their ability to differentiate, as they are capable of differentiating into all cell types in the body except for extraembryonic tissues [6,7]. Pluripotent stem cells include both the naturally occurring embryonic stem cells and the clinically relevant adult induced pluripotent stem cells, which can be engineered and cultured to possess pluripotent capacity [6,9]. Multipotent stem cells are further reduced in potency, restricted to only differentiate into all of the cells of a single cell line, and even more limited are oligopotent stem cells, which are able to divide into only some of the cells in a cell line [6]. Finally, the least potent stem cells are unipotent stem cells, such as unipotent epidermal stem cells, which are restricted to dividing rapidly and only giving rise to a single cell type, with their propensity for division also being of particular interest for use in regenerative medicine therapies [6,9,10]. Muscle satellite cells, for example, have been explored for their regenerative capacity in ameliorating the effects of muscular dystrophies [11,12].

MSCs are generally recognized as fibroblast-like cells with multipotent capacity for differentiation into various connective tissue lineages, chiefly osteoblasts, chondrocytes, and adipocytes [13,14,15]. MSCs constitute a largely heterogeneous population of cells, meaning that different MSCs belonging to the same culture will commit themselves towards differentiation into different cell lineages; however, it does not alone contribute to their efficacy [16,17]. Animal models have shown promise for the use of MSCs through both their restorative and immunomodulatory properties [7,18]. MSCs are particularly desirable because of their immunosuppressive effects, chiefly their ability to suppress T-cell proliferation [19]. As a result, they have emerged as a potential therapeutic in preventing complications related to acute graft-versus-host disease (GVHD) following hematopoietic stem cell transplantation [19,20,21,22]. While variability in protocols exists, clinical results in children have consistently shown MSCs to be an effective treatment for GVHD, with certain MSC-based treatments being approved for pediatric use when corticosteroids prove ineffective [20,22]. Outside of steroid-resistant GVHD, outcomes for MSC treatment have been shown to be effective in tissue repair and enabling engraftment [19,20]. In the context of ischemic strokes, MSCs can potentially aid regeneration by secreting key mediators such as vascular endothelial growth factor (VEGF) and fibroblast growth factor (FGF) to induce angiogenesis and reduce inflammation [18]. Despite this mechanistic underpinning, continued work must be done to understand the properties of MSCs before consistent success can be seen at the level of human clinical trials [7].

Given the wide degree of MSC potency, advances must first be made in optimizing cell tissue origin selection, harvesting, and culturing [7,18]. Regarding tissue origin, data from clinical trials are still incongruous on whether to utilize bone marrow-derived MSCs (BM-MSCs) versus adipose tissue-derived stromal cells—both of which have merit based on clinical trials—or when to even consider less-studied sources such as umbilical cord-derived MSCs [7]. Moreover, consideration must be given towards determining when it is best to administer an MSC for the purposes of regeneration. In the case of ischemic stroke, most preclinical studies have suggested treatment while still in the acute stage, although some conflicting evidence in clinical studies has pointed towards effective transplantation during the chronic stage [23]. Investigating these aspects of MSC properties and applicability can aid our understanding of what is most effective in specific contexts. For stroke, in particular, finding ways to optimize the angiogenic and immunomodulatory aspects of a given transplantation would be the most important aspects to consider in terms of treatment efficacy.

Due to the promising safety profile of mesenchymal stromal cells (MSCs) in blood-borne diseases, coupled with the cells’ multi-faceted therapeutic mechanisms, such as angiogenic, neurogenic, and vasculogenic, among other regenerative features, MSC transplantation for stroke has been translated from the lab to the clinic [24,25]. MSCs can influence the paracrine system to produce factors that promote microglia activation, increase astrocyte survival, and promote the bystander effect [25,26,27]. The inflammatory response following a stroke can be reduced by MSC’s immunomodulatory effect, a main driving factor in functional recovery and patient outcomes [25]. MSC therapy is a frontrunner in a clinical setting since, as a vial therapy, it can be feasible, readily accessible, and safe for patients, as demonstrated by preceding clinical trials [24,25]. While preclinical models have shown efficacy, this has not carried over into clinical trials, leaving much to be discovered on MSC administration and viability [26]. Preclinical trials lack understanding in comorbidities associated with stroke, such as obesity and hypertension, which may contribute to the lack of efficacy [23,28]. This disconnect may be decreased with priming effects and standardization for clinical administration and methodology [24,29,30]. Another explanation for this disconnect may stem from differences in translating the results of animal models to human models, such as immunological function and viability. Due to differences in a model’s biology, bench research results may not always predict the efficacy of human clinical trials [31,32].

## 3. Controversies Surrounding MSCs

Despite promising MSC-based therapies, the fundamental biology of MSCs remains poorly understood. This, in turn, limits the clinical utility of MSCs in brain injury and stroke treatment. For instance, it is unclear whether MSCs should be classified as stromal cells or stem cells. Stromal cells form an organ’s supporting architecture but are distinct from the organ components involved in organ function. Stem cells are totipotent progenitor cells that can renew themselves and differentiate into multiple lineages [33]. While MSCs exhibit stem cell attributes, such as self-renewal in culture and multipotency in the mesodermal cell lineage, and can differentiate into mesodermal cell types and undergo chondrogenesis, adipogenesis, and osteogenesis; permanent cell lineage repletion in vivo has led some to argue that MSCs do not meet the necessary criteria for a stem cell and should instead be classified as stromal cells [34].

One of the obstacles to defining MSCs as either stem cells or stromal cells is the paucity of in vivo studies on MSC differentiation [35]. Up to this point, most of the stem cell properties of MSCs have been observed in in vitro systems and, when produced ex vivo, constitute a heterogeneous cell population in which only a fraction of the cells displays self-renewal potential and multipotency. This led the International Society for Cellular Therapy to release a statement in 2005 describing the term mesenchymal ‘stem cell’ as “scientifically inaccurate” and “potentially misleading to the lay public” [36]. While this paper will not take a stance on this decade-long semantic debate, it is clear that MSCs display limited “stemness.” It is relevant here to discuss the clinical consequences of this limited stemness on stroke.

### 3.1. MSC Limited Stemness

The limited stemness of MSCs is one of the key caveats for the clinical application of MSCs. Since MSCs are not embryonic in origin, they display finite divisions and may not fully recapitulate the true definition of stem cells. This caveat means that MSCs have relatively limited stem cell proliferation, migration, and differentiation capacity, which could, in turn, limit their clinical application [17]. Several laboratories have indicated stemness-related gene clusters in undifferentiated and de-differentiated MSCs [37,38]. These stemness-related gene clusters are primarily involved in proliferation, differentiation, and migration [39]. Expression of these genes was significantly decreased once MSCs differentiated into osteoblasts, chondrocytes, and adipocytes. Serial passaging appears to decrease the expression of stemness genes, as well as increase senescence [40]. Since amplification must occur at a large scale to produce sufficient numbers of MSCs for clinical trials, MSC senescence and modifications in gene expression limit the clinical application of MSCs for stroke.

There are several ways this issue of limited stemness might be resolved [17]. One way is to use induced pluripotent stem cell-derived MSCs, which can be passaged 40 times without showing signs of senescence and appear to have increased regenerative capacity in preclinical degenerative disease models compared to tissue-derived MSCs [17,41,42,43]. Another approach is to stimulate the overexpression of sirtuins (SIRT), highly conserved deacylases that play an important role in aging [17]. SIRT3 overexpression in later-passaged cells may restore differentiation capacity and reduce senescence [44]. The ectopic expression of telomerase reverse transcriptase, the introduction of Erb-B2 receptor tyrosine kinase 4, and the knockdown of macrophage migration inhibitory factor are other potential methods for decreasing MSC senescence [17]. More research must be performed to determine whether such approaches can improve the clinical efficacy of MSC therapy for stroke.

### 3.2. Homogeneous vs. Heterogeneous Cell Population Characterization

The paucity of in vivo studies on MSC differentiation produces ambiguities surrounding other aspects of MSC biology as well, such as whether MSCs constitute a homogeneous or heterogeneous cell population. While in vitro experiments produce a heterogeneous population of MSCs, some papers suggest that MSCs are homogeneous in vivo [45]. Confusion surrounding whether BM-MSCs are characterized as a homogeneous or heterogeneous cell population was raised in part to labs using different markers of expanded MSCs to search for MSCs in vivo. The assumption that markers expressed in vitro systems were present in vivo led the scientific community to characterize MSCs as heterogeneous when they are genuinely homogeneous [45]. Another factor that could masquerade MSC heterogeneity is instability, as MSCs are highly proliferative and multipotent cells that differentiate into different cell types based on their environment. In the recent literature, MSCs have been described as a heterogeneous cell population [34]. While MSC samples are highly heterogeneous when cultured under different conditions and can be used to treat conditions ranging from autoimmune disease to bone fractures, clonal analysis dramatically lowers MSC diversity to just a few clones after multiple passages [46].

### 3.3. MSC Cell Replacement vs. Bystander Effects of Secreted Factors

MSCs demonstrate versatility in promoting neurogenesis, which can be utilized as a treatment for various brain injuries, such as stroke. Cell replacement is one mechanism that MSCs can use in ameliorating damage caused by a stroke. MSCs have the ability to differentiate into neurons, which may improve overall neurological function [47,48]. However, one critical issue regarding the differentiation of MSCs is the inconsistency of their propensity to evolve into neuronal cells. This may be modulated by where MSCs are derived from and whether differentiation occurs in vivo or in vitro [49]. As a result, studies have shifted to analyzing other ways MSCs contribute to brain injury recovery. MSCs have demonstrated chaperoning abilities that enhance the brain’s own endogenous repair system [50]. The grafted MSCs may create a bio-bridge that connects the neurogenic niche to the damaged area, facilitating the movement of neuronal stem cells (NSCs) to the target site [50]. These mechanisms are one way that MSCs are able to demonstrate neurogenic effects in damaged brain tissue.

**Figure 1:**
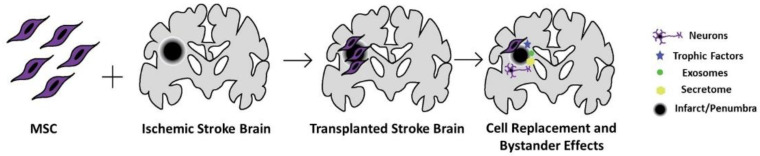
**MSC grafts in the ischemic stroke brain.** MSCs may migrate and repopulate the stroke brain, differentiate to neural cells and release trophic and anti-inflammatory factors and extracellular vesicles, altogether creating a secretome profile of a regenerative brain. While these may not fully repair the lost tissue, MSCs may be able to regenerate the spared tissue (i.e., penumbra and peri-infarct area) surrounding the necrotic infarct core and the adjacent penumbra or peri-infarct area.

The bystander effects of secreted factors from MSCs also have the potential to benefit patients who have suffered from a stroke. A variety of other findings suggest that MSCs’ principal mechanism in promoting neurogenesis is through these secreted factors [26,47,51,52,53]. After transplantation, MSCs release various growth factors to regulate inflammation in areas damaged by stroke [26,51]. Through the bystander effect, the paracrine function of MSCs may enable these growth factors to ultimately support the brain through the promotion of neurogenesis and angiogenesis [26]. MSCs are efficient in the production of extracellular vesicles, which offer another avenue of therapeutic intervention due to their ability to reduce neuroinflammation and promote neurogenesis (Figure 1) [47,53]. Stroke rats treated with MSCs and stroke rats that were treated with MSC-derived extracellular vesicles displayed similar functional recovery levels [54]. These extracellular vesicles are able to cross the blood-brain barrier due to their small size and can deliver various proteins, lipids, and nucleic acids to modulate various processes after a stroke, like inhibiting apoptosis [47]. The key behind axonal growth after treatment with MSC-derived extracellular vesicles involves the transfer of therapeutic miRNA between cells, promoting increased neural plasticity [55,56]. Another advantage of the MSC secretome includes its safety profile in that the extracellular vesicles are unable to self-replicate, which mitigates the threat of cancer from treatment [57,58]. The secretome also releases anti-inflammatory factors, cytokines, and chemokines, which give it the ability to directly modulate the immune response to a damaged site. Furthermore, the content packed into the extracellular vesicles responds to the surrounding microenvironment by communicating with neighboring cells and targeting specific tissues via the expression of integrin subunits [59]. Extracellular vesicles present the opportunity to create a cell-free therapy and have the added benefit of being at low risk for rejection by the immune system [53]. Ultimately, MSCs present many opportunities to assist in the amelioration of damaged neuronal tissue through its paracrine effects that promote neurotrophic factors.

With an understanding of MSCs’ various capabilities to heal or protect neuronal tissue, its applications to patients with stroke are numerous. Bone marrow stromal cells may exhibit neuroprotective effects by reducing ischemic boundary zone scarring and increasing overall proliferating cells, leading to a significant cognitive function recovery in MCAO rats [60]. MSCs may also target the site of ischemic stroke and mitigate permanent damage via brain-derived neurotrophic factor (BDNF), which could affect the regulation of calcineurin, which is a common and problematic issue in stroke pathology [25,60]. Total inhibition of this phosphatase leads to complications like organ intoxication, but overexpression leads to apoptosis in neuronal tissue [25]. There is a lack of literature that has studied potential inhibitors of calcineurin that also minimize organ intoxication [61]. BDNF has been shown to reduce calcineurin activity via the regulation of calcium channels in MCAO [61].

### 3.4. Route, Dose, and Timing of MSC Administration

A significant factor in MSC success is dependent on the dosage, delivery route, and optimal timing. Choosing a delivery route for a therapeutic option is incredibly important, as the placement determines what these stem cells turn into and what other cascades are triggered. Current research places intraparenchymal delivery as the most effective route of administration for MSC in stroke patients, which generates the most significant number of MSCs in the infarct area, leading possibly to the best neurological improvement [62]. On the other hand, a neurosurgical operation may not be well tolerated by all stroke patients and could lead to severe complications [62]. Furthermore, intracerebral transplants limit the number of cells that may be transplanted in order to avoid a mass effect of the brain, whereas systemic approaches do not and provide a greater number of cells [58]. Intra-arterial administration is a practical and minimally invasive strategy, in which more transplanted cells reach the infarcted area with a lower risk of entrapment when compared to the intravenous route [63,64]. Likewise, intravenous delivery is an effective and less invasive route of administration that avoids serious adverse effects associated with more invasive alternatives; however, fewer cells reach the infarcted region due to entrapment primarily in the lungs and spleen, necessitating high cell numbers and posing the risk of pulmonary embolism or thrombosis due to cell accumulation [63,65,66]. Additionally, phase I/II studies found significant statistical and functional improvements in stroke subjects after IV infusion of MSCs throughout a 12-month follow-up period [67]. Another administration route is intranasal delivery, a new way of transplantation with significant convenience and positive outcomes in animal studies; nonetheless, despite its great potential, further studies are needed to evaluate its safety and effectiveness [62]. Perhaps systemic routes such as intra-arterial and intravenous should be considered, as they are less invasive and less likely to cause adverse effects.

The therapeutic dose range for intravenously transplanted MSCs has been shown to be around 4 million cells in 250 g rats, which amounts to over 840 million cells in a 75 kg person [68]. The dose of MSCs required to generate substantial functional and histological improvement in post-ischemic models in animal tests was determined to be 1 × 10^6^ [69]. Similarly, greater motor recovery and infarcted volume reduction were seen in post-ischemic mice treated with 1 × 10^6^ vs. 1 × 10^5^ and 5 × 10^5^ cells [69]. A higher dose of 3 × 10^6^ cells was found to reduce infarct size by 20% and exhibit superior neurological recovery when compared to a 1 × 10^6^ group [62]. Consequently, higher doses may be associated with better outcomes in stroke subjects. In contrast, a high dose (4 × 10^6^) of MSCs injected intravenously into mice with 24-h MCAO did not result in a greater reduction of the infarct area when compared to a lower dose group (1 × 10^6^) [70]. Additionally, 5 × 10^6^ cells were found to be the safest limit for intra-arterially injected MSCs in rodent stroke models, with higher doses linked to significant cell-related side effects [63,64]. Systemic administration of high dosages of MSCs is associated with the risk of microvascular obstruction or embolus formation, which in turn decreases perfusion to the brain and other organs [62]. High dosages of BM-MSCs were associated with a better result in stroke patients in phase II/III clinical trials, with a tendency toward decreased disability in the high-dose group [71]. An ideal injection threshold of 310 × 10^6^ cells was identified as a satisfactory result with no or modest impairment [71]. Furthermore, a meta-analysis considered stem cell therapy to be more effective with high doses of cells and when the intra-arterial route was used [71]. However, there is some evidence of a strong negative association between cell dosage and disability in intravenous patients, indicating that combining high doses of cells with an intra-arterial route is critical for improving neurological outcomes in stroke patients [71]. Additional research is needed to further examine the optimal cell doses and routes of delivery while taking safety and efficacy into account.

An administration time of both 0–6 h and 2–7 days demonstrated superiority in comprehensive neurological functions, suggesting that those times may be the ideal timing for administration [62]. Furthermore, the 0–6 h group showed a greater significant improvement in sensorimotor outcomes [62]. MSCs can reduce infarct volume days to weeks after stroke; the reduction is larger when provided early, within the first 8 h following stroke onset [72]. Therefore, while early administration within 7 days after stroke may be the ideal time for treatment, a narrower time frame might be more beneficial.

### 3.5. Therapeutic Efficacy of the MSC Secretome

Even though MSCs’ migration towards the site of injury is hampered by the hurdles presented in the preferred delivery methods, functional improvements still occur independently as a result of the bystander effect and complementary mechanisms that promote brain repairment and neurological improvement. MSCs exert their therapeutic efficacy in part by producing secretomes, which exhibit anti-inflammatory, immunomodulatory, anti-apoptotic and angiogenic properties, as well as the ability to cross through the blood-brain barrier [55]. In vitro, techniques such as molecular priming, hypoxic preconditioning, tissue engineering, and growth medium composition are used to improve the secretome’s reparative ability [26]. In order to increase the clinical utility of MSCs for stroke, the heterogeneity of MSC populations must be overcome. One potential solution to this is to prime MSCs with certain conditions in order to make them express a particular desired phenotype. Over the past few years, inflammatory cytokines or mediators, hypoxia, chemical agents, and other molecules have been used to prime MSCs [30]. This results in an increase in therapeutic efficacy due to increased immunosuppressive activity, increased secretion of anti-inflammatory factors, improved engraftment, and upregulation of angiogenic activity [30].

Enhancing MSCs with priming effects may improve their durability and efficacy in a hostile environment by engineering these cells to express specific signals or activators to promote lineage differentiation [30]. Priming with pro-inflammatory cytokines aims to support immunosuppressant functions and increase immunomodulatory factors, but their preservation depends on the different tissue sources of MSCs [30,73]. Specific therapeutic inflammatory cytokines may increase regeneration and stimulate an immune response conducive to brain repair [26,74]. Molecular priming may boost the efficacy of MSC treatments by providing a cell population with qualities that allow them to respond better to the ischemia and inflammatory milieu post-stroke [26]. In vivo research on MSC priming in stroke is limited. Notably, IL-1-alpha-primed MSCs at the time of reperfusion results in a significant neuroprotective effect in a mouse model, which may be due to IL-1alpha driving the MSC secretome into a more anti-inflammatory, prototrophic phenotype [75,76,77]. However, despite its exciting potential, there are concerns that priming may increase the immunogenicity or tumorigenicity of MSCs; thus, more research must be done in order to see whether such priming is safe and effective in human trials [30].

In vitro hypoxia preconditioning of MSCs enhances its therapeutic effects via upregulation of the expression of pro-survival genes, secretion of cytokines and trophic factors, angiogenic mediations, inflammatory mediators, and promotion of multipotency [74,78]. Hypoxic priming attempts to replicate the target cellular conditions and can improve angiogenic capacity under ischemic insult [30,79]. In particular, hypoxic priming using serum deprivation increases endothelial cell proliferation of the vascular endothelium [26].

MSCs grown in 3D culture may also promote angiogenesis and reduce inflammation, which may repair damaged tissues in ischemic conditions [30,79]. Overall, 3D MSC cultures enhance MSCs’ anti-inflammatory properties, augment their tissue regenerative and reparative effects with improved angiogenesis, facilitate the differentiation potentials of multiple lineages, improve MSC stemness and posttransplant survival, and slow in vitro replicative senescent processes [80]. The injection of 3D MSCs in MCAO rats exhibited a significant reduction of the volume of the ischemic injury, improvement in functional recovery, and improved sensorimotor outcomes, which may be attributed to the technique’s ability to restore the expression of homing receptors of MSCs and enhance the secretion of factors mediating the inflammatory and immune response [25]. Modifying MSCs’ secretome to improve their therapeutic potential through a conditioned medium or serum preconditioning, such as endothelial growth medium, is a viable strategy when a specific environment must be mimicked or when the molecule targeted to elicit a specific effect is unknown [26].

Extracellular vesicles derived from MSCs (MSC-EV) have been shown to be beneficial in stroke repair by promoting functional recovery and increased plasticity [26]. This may be due to the role of MSC-EVs in delivering miRNA to the damaged cell through targeting or signaling [81]. MSC-EVs in ischemic damage may induce neurogenesis, white matter remodeling, and angiogenesis [26]. To advance clinically, rigorous efforts are warranted to achieve strict standardization, quality control production, phenotypic characterization, and precise signaling or targeting to specific sites of MSC-EVs. Techniques used to enhance the secretome’s therapeutic potential might prove beneficial as a technique to improve a stroke patient’s outcome, regardless of MSC migration obstacles, and because it drives the effect towards a more anti-inflammatory and pro-angiogenic model [25,26]. To this end, protocols involving the cell secretome from the same source must be standardized across the field to limit result variability [74,82].

## 4. Conclusions and Future Directions

**Figure 2:**
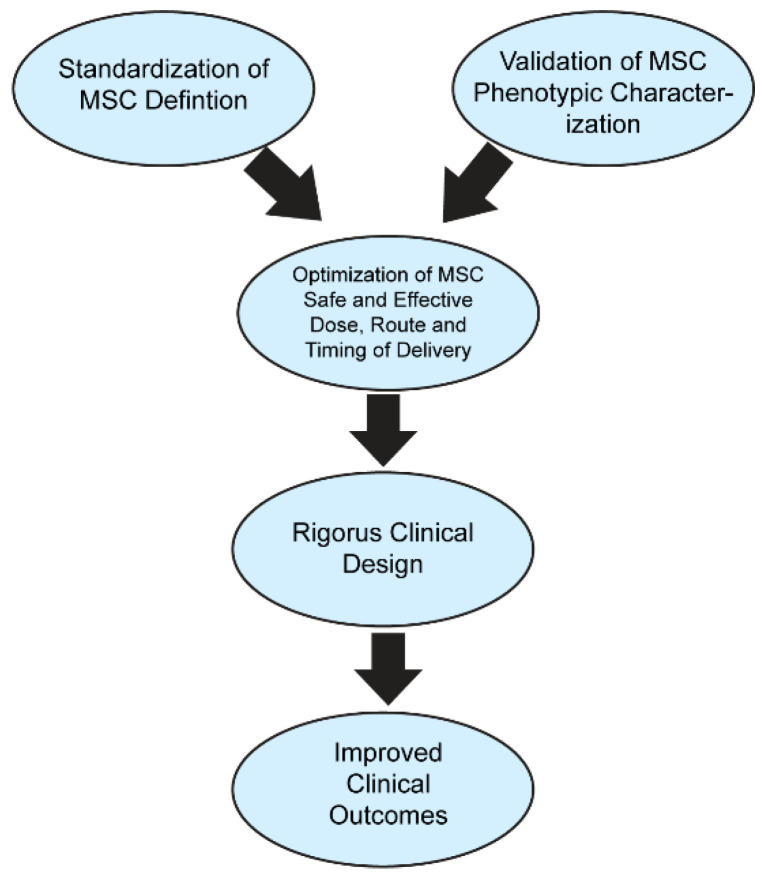
**MSC standardization and optimization leading to improved functional outcomes.** The standardization of the treatment and classification of MSCs combined with the standardization of MSC administration can increase their clinical applications in clinical trials and settings, which may lead to improved functional outcomes.

MSC may be a novel treatment option for ischemic brain injury, as shown in pre-clinical models. However, while laboratory data generally support the application of MSCs in stroke patients, the clinical outcomes are mostly relegated to their safety profile, with the efficacy of the therapy still elusive. Due in part to the ambiguity surrounding the definition of MSCs, this has resulted in a negative impact on the field’s development, generating confusion and counterproductivity. The International Society for Cell & Gene Therapy Mesenchymal Stromal Cell (ISCT MSC) considers a set of characteristics and properties as prerequisite criteria to obtain the proper definition of MSCs, including a plastic adhesion property, the expression of CD73, CD90, and CD105, the lack of expression of CD11b, CD14, CD19, CD34, CD45, CD79a and HLA-DR, and the in vivo capability to differentiate into adipocyte, chondrocyte, and osteoblast lineages [83]. The definition of stem cells must encompass their ability to be unlimited self-renewal cells with the capability of developing into cells from diverse lineages, present in several locations throughout the body and classified as embryonic, fetal, and adult stem cells [84]. MSC are multipotent as well, being present as a distinct but rare population of the tissue where they are found, such as bone marrow, the umbilical cord, and adipose tissue, with significant secretory, immunomodulatory, and homing capabilities as a large population [57,83,84]. For the future success of the field, a suitable and accepted definition, characterization, and complete understanding of the mechanism of action must be specified and supported across all future MSC studies. Optimizing MSC administration via priming effects and an enhanced secretome, as well as standardizing clinical applications, may present improved functional outcomes in ischemic stroke patients (Figure 2).

## Data Availability

Not applicable.
